# Polycyclic Aromatic Hydrocarbons (PAHs) in the Environment: Occupational Exposure, Health Risks and Fertility Implications

**DOI:** 10.3390/toxics13030151

**Published:** 2025-02-23

**Authors:** Luigi Montano, Giorgio Maria Baldini, Marina Piscopo, Giovanna Liguori, Renato Lombardi, Maria Ricciardi, Gennaro Esposito, Gabriella Pinto, Carolina Fontanarosa, Michele Spinelli, Ilaria Palmieri, Daniele Sofia, Carlo Brogna, Cosimo Carati, Mauro Esposito, Pasquale Gallo, Angela Amoresano, Oriana Motta

**Affiliations:** 1 Andrology Unit and Service of Lifestyle Medicine in Uro-Andrology, Local Health Authority (ASL) Salerno, 84124 Salerno, Italy; 2 Coordination Unit of the Network for Environmental and Reproductive Health (Eco Food Fertility Project), Oliveto Citra Hospital, 84124 Salerno, Italy; 3 Unit of Obstetrics and Gynecology, Department of Interdisciplinary Medicine (DIM), University of Bari Aldo Moro, 70121 Bari, Italy; gbaldini97@gmail.con; 4 Department of Biology, University of Naples Federico II, Via Cinthia 26, 80126 Naples, Italy; marina.piscopo@unina.it; 5 Territorial Pharmaceutical Service, Local Health Authority (ASL), 71121 Foggia, Italy; giovanna.liguori@aslfg.it (G.L.); renato.lombardi@aslfg.it (R.L.); 6 Department of Chemistry and Biology, University of Salerno, Via Giovanni Paolo II 132, 84084 Fisciano, Italy; mricciardi@unisa.it; 7 UOSM Nola ASL Napoli 3 Sud, 80035 Napoli, Italy; genesp@libero.it; 8 Department of Chemical Sciences, University of Naples Federico II, Via Cinthia 26, 80126 Naples, Italy; gabriella.pinto@unina.it (G.P.); carolina.fontanarosa@unina.it (C.F.); michele.spinelli@unina.it (M.S.); angela.amoresano@unina.it (A.A.); 9 INBB-Istituto Nazionale Biostrutture e Biosistemi, Consorzio Interuniversitario, 00136 Rome, Italy; 10 Department of Environmental, Biological and Pharmaceutical Sciences and Technologies, University of Campania Luigi Vanvitelli, 81100 Caserta, Italy; ilaria.palmieri@unicampania.it; 11 Research Department, Sense Square Srl, 84084 Salerno, Italy; dsofia@unisa.it; 12 Department of Computer Engineering, Modeling, Electronics and Systems, University of Calabria, Via P. Bucci, Cubo 44/a Rende, 87036 Arcavacata, Italy; 13 Department of Research, Craniomed Group Facility Srl, 20091 Bresso, Italy; dir.brogna@craniomed.it; 14 Student of Department of Medicine Surger, University Cattolica Sacro Cuore, Largo Francesco Vito, 1, 00168 Roma, Italy; cosimocarati@gmail.com; 15 Istituto Zooprofilattico Sperimentale del Mezzogiorno, Dipartimento Coordinamento di Chimica, Via della Salute, 2, 80005 Portici, Italy; mauro.esposito@izsmportici.it (M.E.); pasquale.gallo@izsmportici.it (P.G.); 16 Department of Medicine Surgery and Dentistry “Scuola Medica Salernitana”, University of Salerno, Via S. Allende, 84081 Baronissi, Italy; omotta@unisa.it

**Keywords:** polycyclic aromatic hydrocarbons, environment, occupational exposure, bioaccumulation and metabolization, health effects, fertility

## Abstract

Polycyclic aromatic hydrocarbons (PAHs) are a group of organic compounds with fused aromatic rings, primarily derived from combustion processes and environmental pollutants. This narrative review discusses the most relevant studies on PAHs, focusing on their sources, environmental and occupational exposure, and effects on human health, emphasizing their roles as carcinogenic, mutagenic, and teratogenic agents. The primary pathways for human exposure to PAHs are through the ingestion of contaminated food (mainly due to some food processing methods, such as smoking and high-temperature cooking techniques), the inhalation of ambient air, and the smoking of cigarettes. Coke oven workers are recognized as a high-risk occupational group for PAH exposure, highlighting the need for appropriate strategies to mitigate these risks and safeguard worker health. PAHs are metabolized into reactive intermediates in the body, which can lead to DNA damage and promote the development of various health conditions, particularly in environments with high exposure levels. Chronic PAH exposure has been linked to respiratory diseases, as well as cardiovascular problems and immune system suppression. Furthermore, this review underscores the significant impact of PAHs on reproductive health. The results of the reported studies suggest that both male and female fertility can be compromised due to oxidative stress, DNA damage, and endocrine disruption caused by PAH exposure. In males, PAHs impair sperm quality, while, in females, they disrupt ovarian function, potentially leading to infertility, miscarriage, and birth defects. Fetal exposure to PAHs is also associated with neurodevelopmental disorders. Given the extensive and detrimental health risks posed by PAHs, this review stresses the importance of stringent environmental regulations, occupational safety measures, and public health initiatives to mitigate exposure and safeguard reproductive and overall health.

## 1. Introduction

Polycyclic aromatic hydrocarbons are a class of organic compounds composed of two or more fused aromatic rings, with benzene as the fundamental structural unit. These compounds can exhibit linear, clustered, or angular arrangements and may include substituent groups such as alkyl, nitric, or amino groups [1].

Polycyclic aromatic hydrocarbons (PAHs) demonstrate significant stability due to their aromatic structure. They are classified based on their molecular weight into low-molecular-weight (LMW) PAHs, which consist of two or three aromatic rings, and high-molecular-weight (HMW) PAHs, which contain four to six rings. LMW PAHs, such as naphthalene and anthracene, are generally volatile and predominantly exist in the vapor phase in the atmosphere. In contrast, HMW PAHs, such as fluoranthene and chrysene, are found in both the vapor and particulate phases, while PAHs with five or more rings, like benzo[g,h,i]perylene, are mainly associated with particulate matter [2,3].

The United States Environmental Protection Agency (US-EPA) (Table 1) has classified sixteen polycyclic aromatic hydrocarbons as priority pollutants owing to their harmful effects on human health and the environment. Although many PAHs are categorized by the International Agency for Research on Cancer (IARC) as probable or possible human carcinogens, only benzo[a]pyrene has been unequivocally identified as a confirmed human carcinogen [4]. The toxicity of PAHs is shaped by their physical and chemical characteristics, such as high melting and boiling points, low vapor pressure, and poor solubility in water. These factors tend to increase as the molecular weight of the PAH rises [2,3]. They exhibit solubility in organic solvents, lipophilicity, and adherence to organic matter. Their characteristic ultraviolet absorbance spectra and fluorescence, emitting at specific wavelengths upon excitation, facilitate identification. Low-molecular-weight PAHs, due to their limited aqueous solubility, can contaminate groundwater aquifers [5].

**Figure 1:**
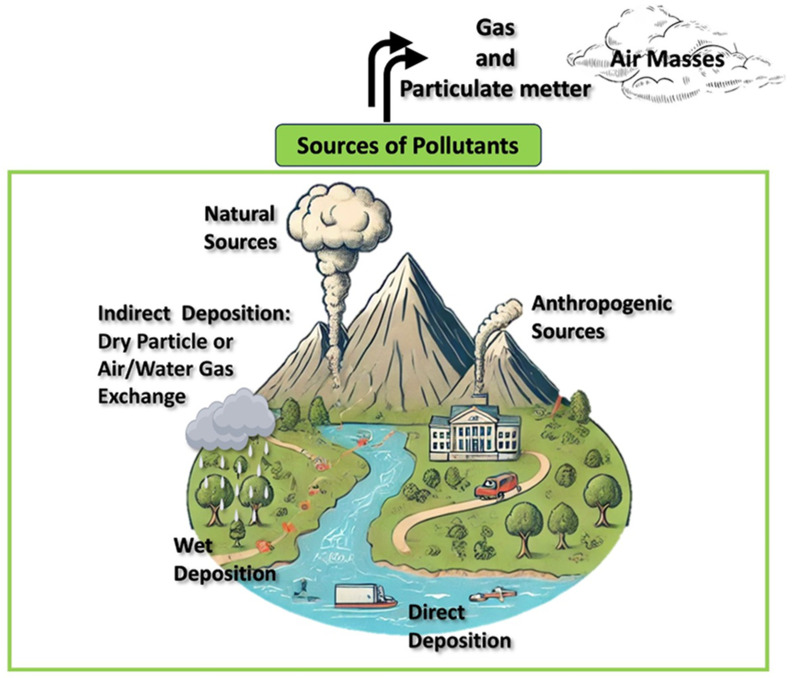
Sources and pathways of PAHs in the environment.

PAHs are primarily produced when organic materials like coal, wood, and petroleum undergo incomplete combustion or pyrolysis at temperatures between 350 °C and 1200 °C [6,7]. Their formation is considered a complex process, involving the repolymerization of hydrocarbon fragments created during cracking. PAHs can come from both natural sources and human activities. Once in the atmosphere, they can travel both short and long distances, with higher deposition rates typically seen in urban areas compared to rural ones [8,9]. PAHs are eliminated from the atmosphere through dry and wet depositional processes. Dry deposition takes place via gravitational settling, while wet deposition entails the removal of PAHs by precipitation [10,11,12]. Their deposition rates are influenced by meteorological factors such as the temperature and wind speed, as well as the presence and type of vegetation. Vegetation, particularly tree crowns, can effectively capture particulate matter (Figure 1) [13,14].

**Figure 2:**
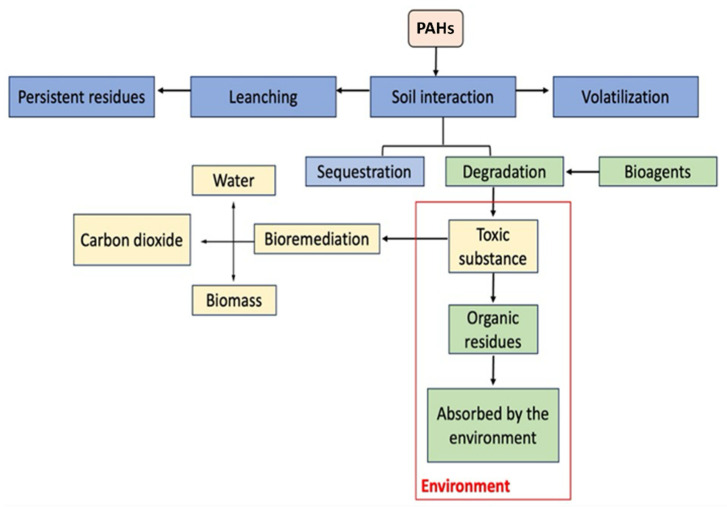
Fate of PAHs in soil (adaptation from Stokes et al., 2005 [19]).

PAHs, frequently detected as chlorinated derivatives of compounds such as naphthalene and phenanthrene, are present in tap water at concentrations ranging from 0.1 to 1.0 ng/L [15]. PAHs can attach to airborne particles, making it easier for them to enter the respiratory and circulatory systems, particularly when the particles are smaller than 2 µm in diameter [16,17,18]. In terrestrial environments, PAHs accumulate in soil due to their lipophilic properties and persistence. These compounds undergo various environmental processes, including volatilization, adsorption, and leaching (Figure 2).

Additionally, PAHs can enter and move through the food chain when taken up by plants and animals [19,20,21,22]. PAHs are persistent environmental pollutants, but they can break down through abiotic processes like photodegradation and microbial activity. Soil microorganisms can adapt to metabolize these compounds, which helps in their biodegradation [23,24,25,26,27,28].

PAHs are broken down by a variety of organisms, with white-rot fungi (Basidiomycetes) being particularly significant in their decomposition. Other fungal genera, such as *Fusarium* and *Aspergillus*, also aid in PAH degradation through the action of specialized enzymes [29,30]. A collaborative degradative process involving both fungi and bacteria can result in the complete mineralization of PAHs. In this mutualistic interaction, fungi convert PAHs into more water-soluble metabolites, which are then further mineralized by bacteria [31,32,33]. Bioremediation is a process that uses microorganisms, including fungi and bacteria, to break down harmful compounds into harmless substances like CO_2_ and water, providing an effective means of cleaning contaminated soil and aquifers (Figure 2). During bioremediation, bacteria gradually break down toxins, with their populations decreasing as the contaminants are exhausted. In bacteria, PAH degradation typically occurs through aerobic metabolism, where dioxygenase enzymes are used to oxidize the benzene rings, creating cis-dihydrodiols, which are further metabolized into CO_2_ and water [34,35,36,37]. Ligninolytic fungi release enzymes like laccase and peroxidase, which facilitate the oxidation of PAHs. This enzymatic oxidation produces free radicals that trigger the breakdown of PAHs into quinones, which are subsequently mineralized by bacteria [38,39,40,41].

This narrative review critically examines polycyclic aromatic hydrocarbons (PAHs), focusing on their sources, exposure pathways, health risks, and environmental persistence. PAHs, prevalent in both natural and anthropogenic environments, pose significant health concerns, especially in industries like coal mining, asphalt production, and petrochemical processing, where occupational exposure is elevated. Chronic exposure to PAHs has been linked to a range of serious health issues, including various cancers, respiratory and cardiovascular diseases, reproductive health problems, immunosuppression, and endocrine disruption [42,43,44,45,46,47]. PAHs exert toxicity through mechanisms such as oxidative stress, DNA damage, and endocrine disruption, further complicating their health impacts [5,48]. Experimental research plays a pivotal role in elucidating the mechanisms through which PAHs affect human health. For example, long-term exposure to PAHs in animals has been shown to induce various cancers, such as lung, stomach, and skin cancer, depending on the route of exposure (inhalation, ingestion, and dermal contact, respectively) [49]. Additionally, the embryotoxic effects of PAHs such as benzo(a)anthracene, benzo(a)pyrene, and naphthalene have been observed in animal models, with studies showing that high exposure to benzo(a)pyrene during pregnancy resulted in congenital disabilities and reduced body weight in offspring [50]. Cardiovascular effects have also been identified in animal studies, although challenges in extrapolating these findings to humans exist, as rodents typically do not develop atherosclerosis as humans do, and the plaques in commonly used mouse models do not rupture as they do in humans [51]. Moreover, PAHs like phenanthrene, a weak aryl hydrocarbon receptor (AHR)-mediated CYP-1 activator, have been shown to induce cardiac hypertrophy in animals through mechanisms such as decreased miR-133 expression, affecting cardiomyocytes’ size and protein content [52].

This narrative review also discusses the persistence and bioaccumulation of PAHs in the environment, highlighting their detrimental effects on human fertility and broader health concerns. Their ability to accumulate in environmental matrices such as air, soil, water, and food chains intensifies the long-term public health risks posed by these pollutants [42]. By addressing the sources, distribution, degradation, and toxicological mechanisms of PAHs, this review provides essential insights for the development of regulatory measures and preventive strategies to mitigate their impacts on both human health and the environment. Ultimately, understanding the complex relationship between PAHs, their persistence in the environment, and their toxic effects is crucial in reducing the associated health risks and informing future public health and environmental policies.

## 2. Materials and Methods

This narrative review was conducted to summarize the current understanding of PAHs in the environment, with an emphasis on occupational exposure, human health effects, and impacts on fertility. A thorough literature search was carried out across three major scientific databases: Google Scholar, PubMed, and MEDLINE. The data collection spanned from 1992 to 2024, focusing on peer-reviewed articles published in English.

To ensure the relevance and accuracy of the information, a set of predefined search terms was employed, including “polycyclic aromatic hydrocarbons”, “PAHs human health”, “PAH exposure”, “PAHs cancer”, “PAHs immune system”, “PAHs occupational exposure”, “PAHs environmental risk”, “PAHs infertility”, “PAHs IVF/ICSI”, “PAHs male infertility”, “PAHs sperm quality”, and “PAHs female infertility”.

The inclusion criteria were limited to studies published in peer-reviewed journals that focused on human health, environmental exposure, or regulatory measures related to PAHs. Articles discussing animal studies, ecosystem effects, or biomonitoring related to PAH exposure were excluded unless they were directly relevant to human health impacts.

This approach was intended to provide a thorough evaluation of the sources, health risks, and environmental impacts of PAHs, with the goal of aiding in the development of effective regulatory strategies and preventive actions to reduce exposure.

## 3. Exposure to Polycyclic Aromatic Hydrocarbons

### 3.1. Environmental Exposure from Air, Water, Soil, and Food

**Figure 3:**
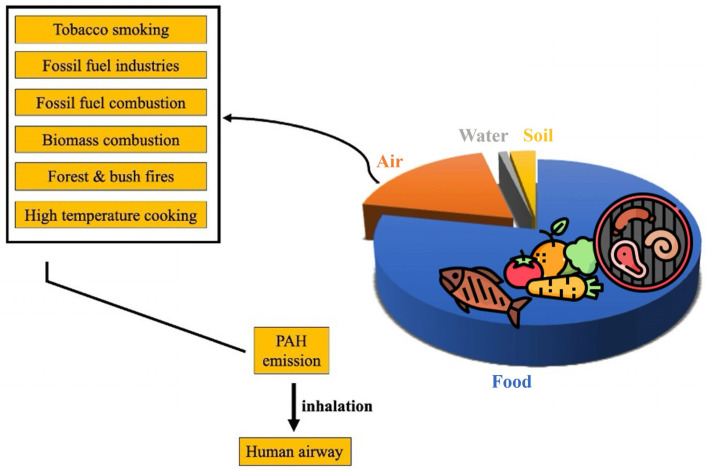
Main sources of human exposure to PAHs, including food, air, water, and soil [53]. On the left, the various sources of PAHs in the air are outlined in detail.

PAHs demonstrate moderate persistence in the environment and are prone to bioaccumulation. The concentrations of PAHs in aquatic organisms, such as fish and shellfish, are expected to be significantly higher than in the surrounding environment. Bioaccumulation has also been noted in terrestrial invertebrates. However, the activity of metabolic processes is adequate to prevent biomagnification [42]. Given the ubiquitous nature of polycyclic aromatic hydrocarbons as environmental contaminants, pervasive across all environmental matrices, humans are exposed to these compounds through various pathways, including inhalation, dermal contact, and ingestion via air, water, soil, and food (Figure 3).

The primary pathways for human exposure to PAHs are through the ingestion of contaminated food, the inhalation of ambient air, and the smoking of cigarettes or exposure to smoke from open fireplaces. Although there may also be exposure to PAHs through contaminated water and soil, these exposure routes can be considered of lesser importance than those mentioned above, as any contamination of these compartments, in the majority of cases, results in the contamination of food for human consumption, thus falling into the food category. The inhalation of PAHs occurs through various sources, including tobacco smoke, the combustion of fossil fuels and biomass, and high-temperature cooking. Among the myriad chemical constituents of cigarette smoke, PAHs represent a significant threat to human health. Tobacco is estimated to contain 100 ng or more of total PAHs per gram, and smokers inhale approximately 0.26 μg of benzo[a]pyrene per pack of 20 cigarettes [54,55].

For non-smokers, the main means of exposure to PAHs is through consuming contaminated food. PAH contamination in food can come from both environmental sources and food processing methods. In unprocessed foods, PAHs mainly result from environmental pollution, such as particulate matter from the air settling on crops like wheat, fruits, and vegetables; absorption from contaminated soil by root vegetables like potatoes and carrots; and bioaccumulation in aquatic organisms such as fish, mollusks, and crustaceans from polluted waters. In processed foods, common sources of PAHs include high-temperature cooking techniques like grilling, frying, baking, and toasting, as well as certain manufacturing processes, particularly drying and smoking [56].

PAHs in food can be categorized as “endogenous” PAHs, generated through the pyrolysis of carbohydrates, lipids, and proteins during high-temperature cooking processes, and “exogenous” PAHs, originating from fuel combustion during cooking or smoking. Food processing techniques such as drying and high-temperature cooking methods (e.g., grilling, roasting, frying) are significant sources of PAH contamination in food [57,58,59]. Grilling, specifically, can generate PAHs through the incomplete combustion of lipids that fall onto the heat source [60]. The literature indicates a strong correlation between the PAH concentrations in grilled meat, their fat content, and their proximity to the heat source. In smoked fish and meat products, the PAH levels can reach up to 200 μg/kg, while grilled meat can contain approximately 130 μg/kg of PAHs. The baseline PAH levels in uncooked foods typically range from 0.01 to 1 μg/kg [42].

Contemporary research demonstrates that, in grilled meat products, direct exposure to flames results in substantial PAH formation, with the benzo[a]pyrene concentrations reaching up to 200 µg/kg. Conversely, embers emit considerably smaller quantities (1–20 µg/kg) of benzo[a]pyrene. Exogenous PAHs, generated from fuel combustion, can contaminate food surfaces through the combustion gases and fumes. The fuel type and smoke generation conditions play a crucial role, particularly in smoking processes. 

Dietary intake, including the consumption of cereals, vegetables, fruits, meat, fish, oils, tea, and coffee, is the main way in which humans are exposed to PAHs [9,61,62,63,64,65,66,67,68]. An analysis of 23 polycyclic aromatic hydrocarbons in various bread varieties revealed total PAH concentrations ranging from 2.61 μg/kg to 43.4 μg/kg, with variations observed between the crust and the crumb [69]. While grilled and smoked meat and fish contribute to polycyclic aromatic hydrocarbon intake, cereals, fats, and oils represent the primary dietary sources of PAHs. Drying processes and combustion fumes are key factors in PAH contamination, particularly in seed oils and pomace oil production [70,71]. The PAH concentrations are typically elevated in products cultivated in proximity to roadways and urban centers due to vehicular and industrial emissions [72]. Trace quantities of PAHs such as phenanthrene, fluoranthene, and pyrene are ubiquitous in unprocessed fruits and vegetables, with lighter PAHs like naphthalene, acenaphthylene, and acenaphthene also detected in certain varieties [9]. An investigation conducted in Saudi Arabia revealed elevated PAH concentrations in root vegetables such as potatoes (11 μg/kg) and carrots compared to turnips (9.26 μg/kg). Among fruits, the peel exhibited greater contamination than the flesh. Cabbage demonstrated the highest PAH levels among leafy vegetables (8.34 μg/kg). The benzo[a]anthracene concentrations were the highest in turnips (2.21 ± 1.75 μg/kg), while the benzo[e]pyrene levels were the highest in potatoes (2.90 ± 1.10 μg/kg) [73].

Elevated PAH concentrations in leafy vegetables, a significant dietary component for many African populations due to their recognized health benefits, present a substantial health risk to consumers. Various leafy vegetable species were sampled from farms situated along Nima Creek, Accra, Ghana. Varying concentrations of acenaphthene, acenaphthylene, benzo[a]anthracene, benzo[b]fluoranthene, and benzo[a]pyrene were detected, while naphthalene was ubiquitous across all vegetable samples. The mean phenanthrene concentrations in Chinese cabbage varied across different plant tissues, following the order of roots (0.744 ± 0.16 μg/kg) ≥ leaves (0.598 ± 1.21 μg/kg) ≥ stem (0.327 ± 1.01 μg/kg) [74].

Undeniably, both food processing methods (such as dehydration and smoking) and high-temperature cooking techniques (like grilling, roasting, and frying) lead to significant levels of PAHs. Additionally, some crops may either produce PAHs on their own or absorb them from environmental sources like water, air, or soil, contributing to human exposure to these contaminants.

### 3.2. Occupational Exposure

Occupational PAH exposure can arise from the inhalation of exhaust fumes by workers and those engaged in mining, metalworking, or petroleum refining. As early as 1775, occupational skin cancer was linked to soot exposure among London’s chimney sweeps. Soot contains elevated levels of PAHs, which were among the first substances identified as carcinogens [75]. This finding was subsequently corroborated among workers within the paraffin industries of Scotland and Germany.

**Figure 4:**
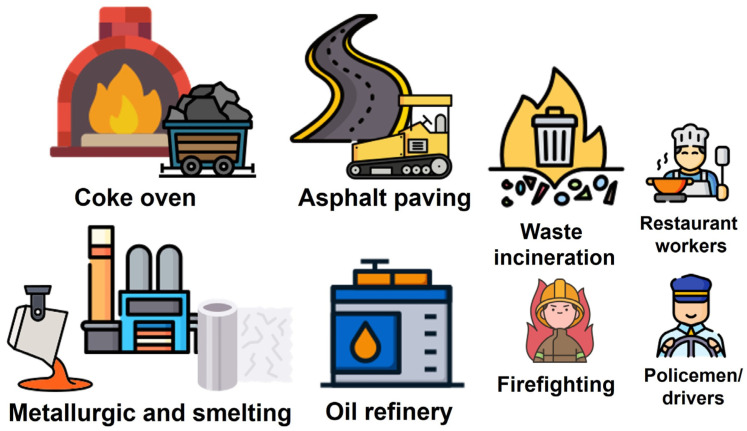
Occupational routes of PAH exposure are shown, arranged from the highest to lowest levels of exposure, from left to right.

Investigations of occupational PAH exposure have predominantly concentrated on industrial sectors such as coke oven production, asphalt/bitumen/road paving, metallurgy, electrode manufacturing, aluminum production and smelting, and oil refining. Secondary areas of focus include non-industrial sectors such as firefighting and waste incineration and, to a lesser degree, restaurant workers, police officers, drivers, air force personnel, groundskeepers, and naval personnel (Figure 4).

Several investigations have explored the potential correlations between airborne PAHs, typically measured using passive air samplers, and biomarkers of exposure. The most commonly employed urinary markers in these studies were 1-hydroxypyrene (1-OH-PYR) and 3-hydroxybenzo[a]pyrene (3-OH-BaP) [76,77]. For instance, within the United States population, a geometric mean 1-OH-PYR concentration of 79.8 ng/L was observed. Adult smokers exhibited urinary 1-OH-PYR levels threefold greater than those of non-smokers [77]. Typically, elevated airborne concentrations correlate with increased urinary metabolite levels in exposed workers. A cross-industry occupational hygiene assessment, conducted to ascertain the exposure levels within United Kingdom industries, revealed an 8 h time-weighted average airborne PAH concentration range of 0.4–1912.6 µg/m^3^. A strong correlation was observed between the 1-OH-PYR levels and airborne benzo[a]pyrene concentrations (0.01–6.21 µg/m^3^) [78]. It is important to acknowledge that PAH exposure involves a complex mixture of compounds; thus, a single metabolite cannot be universally employed to assess exposure to all PAHs. A correlation between the atmospheric and urinary PAH metabolite concentrations is not consistently observed, suggesting that, in certain occupational settings, exposure routes other than inhalation, such as dermal absorption, may be significant [79].

Coke oven workers are recognized as a high-risk occupational group for PAH exposure. Across various coking production sectors, the total airborne PAH concentrations have been measured to be between 12 and 47 µg/m^3^, with benzo[a]pyrene concentrations ranging from 0.05 to 1.05 µg/m^3^. This substantial exposure elevates the risk of PAH-related health complications, including carcinogenesis and respiratory ailments, among coke oven workers, emphasizing the critical need for stringent occupational safety protocols in these environments [80]. While the measured PAH concentrations in coke oven settings fall slightly below the recommended exposure limit of 0.1 mg/m^3^ for a 10 h workday or 40 h workweek, as advised by the National Institute for Occupational Safety and Health (NIOSH) [3], these findings nevertheless raise substantial concerns.

This is particularly relevant in the absence of adequate personal protective equipment (PPE). Even concentrations below the regulatory thresholds can present substantial health risks if workers lack sufficient protection, as chronic PAH exposure, even at low levels, can result in long-term adverse health outcomes, including carcinogenesis, respiratory illnesses, and other toxic effects. The utilization of appropriate PPE is essential in mitigating these risks and safeguarding worker health.

## 4. Effects of Polycyclic Aromatic Hydrocarbons on Human Health

### 4.1. Bioaccumulation and Metabolization of PAHs in the Human Body

From a toxicological standpoint, experimental findings indicate that a crucial determinant of PAHs’ carcinogenicity is the presence of at least four fused rings within their molecular structure. This structural characteristic is a prerequisite, albeit not solely sufficient, for PAHs’ carcinogenicity. The fusion of these rings diminishes their aromatic nature, thereby increasing their susceptibility to metabolic transformations, notably epoxidation. Epoxidation generates reactive intermediates, exhibiting heightened carcinogenic potential. Critically, PAH-derived diol epoxides are the ultimate carcinogenic species. PAHs, in their native forms, generally lack the electrophilic properties required for covalent interactions with nucleophilic sites within DNA (e.g., nitrogen, oxygen, or phosphorus atoms). Consequently, PAHs are not inherently carcinogenic but require metabolic activation within the organism to produce carcinogenic metabolites. These metabolic processes convert chemically inert PAHs into electrophilic intermediates. These highly reactive intermediates can bind to biological macromolecules, including DNA, resulting in mutations and initiating carcinogenesis. Therefore, the carcinogenic potential of PAHs is largely contingent upon their metabolic activation, underscoring the importance of elucidating these biochemical pathways to evaluate the risks associated with PAH exposure [81]. The toxic effects are driven by the formation of reactive intermediates and the activation of a specific receptor known as AHR. First identified in 1976, AHR is a ligand-dependent transcription factor with a helix–loop–helix structure that controls the expression of various genes. In addition to regulating the production of enzymes involved in metabolic processes, AHR plays a crucial role in immune system regulation, stem cell function, cell differentiation and proliferation, apoptosis, carcinogenesis, and drug metabolism [71,72,73,74]. Environmental contaminants such as dioxins or non-halogenated PAHs are the most characteristic classes of ligands for this receptor. While many genes are regulated by AHR, the most extensively studied are those encoding enzymes involved in metabolism, such as the CYP1A1 gene. The induction of CYP1A1 is an AHR-dependent response that has been investigated across various species and serves as a model to understand the mechanism through which AHR regulates gene expression. The toxic substance enters the cell through the plasma membrane, with small or nonpolar molecules typically crossing the membrane via passive diffusion through the lipid bilayer. Once inside, it binds to the cytosolic AHR, which exists as part of a multiprotein complex. Upon binding, AHR is believed to undergo a conformational change that exposes a nuclear localization sequence, allowing the complex to translocate into the nucleus. The ligand–AHR complex then dimerizes with a nuclear protein called the AHR nuclear translocator (ARNT). This process transforms AHR into a high-affinity DNA-binding form. The multimeric complex (ligand–AHR–ARNT) then binds to its specific recognition sites on DNA, known as dioxin regulatory elements (DRE), located upstream of the CYP1A1 gene and other target genes. This multimeric complex then activates the transcription of these genes, leading to the production of mRNAs that encode various proteins and, consequently, enzyme induction. However, while the role of AHR in the biological and toxic effects induced by AHR ligands (such as dioxins and PAHs) is well established, the precise biochemical events responsible for the range of adverse effects caused by these chemicals have yet to be fully understood.

**Figure 5:**
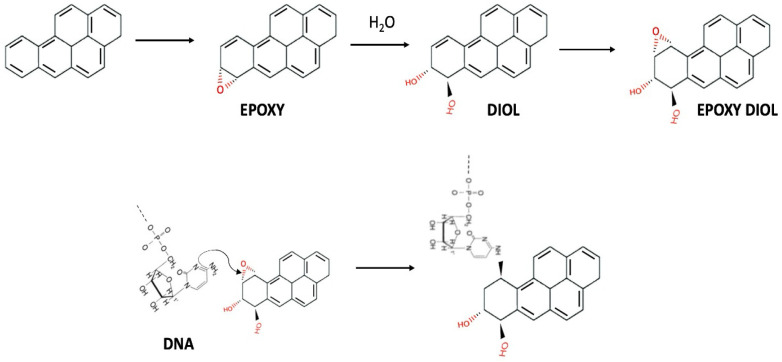
Metabolism of benzo[a]pyrene in mammals [84].

The elimination of PAHs from the body relies on their conversion into water-soluble metabolites, which can be excreted. However, it is the formation of these metabolic products that is responsible for the mutagenic and carcinogenic effects on mammals. Specifically, through an enzymatic reaction mediated by cytochrome P450 monooxygenase, the aromatic rings are oxidized, resulting in the formation of epoxy, diol, and diol-epoxy intermediates [82]. Figure 5 illustrates the metabolism of benzo[a]pyrene in mammals. The initial reaction involves epoxidation at positions 7 and 8, the most reactive sites, resulting in the formation of benzo[a]pyrene-7,8-epoxide. Subsequently, this compound undergoes nucleophilic attack by water, yielding a diol, possessing two hydroxyl groups on adjacent carbon atoms. This diol exhibits increased water solubility, facilitating its elimination, leading to the formation of benzo[a]pyrene-7,8-diol. The newly formed diol can undergo further epoxidation, generating benzo[a]pyrene-7,8-diol-9,10-epoxide, the most potent carcinogenic metabolite, responsible for the mutagenic and carcinogenic effects induced by benzo[a]pyrene. The interaction with DNA is illustrated by the diol epoxide binding to DNA through nucleophilic attack, typically involving the amine groups of purine bases, such as adenine and guanine (Figure 5). Covalent modification by the bulky hydrocarbon moiety introduces significant distortions in the DNA structure, leading to mutations and, consequently, an elevated risk of carcinogenesis. In addition to nucleotide substitutions, other genotoxic effects include frameshift mutations (insertions/deletions of nucleotides within a DNA sequence), S-phase arrest (inhibition of DNA replication during the cell cycle), DNA double-strand breaks, and a wide spectrum of chromosomal aberrations [83].

The metabolic processes of epoxidation and hydration constitute the organism’s attempts to introduce hydroxyl groups into hydrophobic molecules like PAHs, thereby enhancing their water solubility and facilitating their elimination. Not all PAHs induce genotoxic effects, as not all serve as precursors to these reactive intermediates. Specifically, PAHs exhibiting carcinogenic properties typically comprise more than three benzene rings and possess a bay region: a sterically hindered region formed by the angular fusion of benzene rings, characterized by a high electron density [85]. Epoxide diols exhibit notable resistance to enzymatic detoxification. They are poor substrates for epoxide hydrolase and glutathione conjugation, a detoxification mechanism involving the conjugation of xenobiotics with glutathione, a tripeptide composed of glycine, cysteine, and glutamate. The enzyme catalyzing this reaction is glutathione S-transferase. This resistance is putatively attributed to the steric hindrance imposed by the bay region. Scientific investigations have demonstrated that the majority of PAH metabolites are excreted in the feces and urine. Both hydroxylated metabolites and unmetabolized PAHs in urine serve as valuable biomarkers of human exposure [86].

Given the substantial lipophilicity of PAHs, their bioavailability following ingestion or inhalation is considerable. Scientific studies have demonstrated detectable PAH levels in virtually all internal organs, particularly those rich in adipose tissue. These organs can serve as storage depots from which PAHs are gradually released. Upon ingestion or inhalation, PAHs are rapidly absorbed through the gastrointestinal tract or pulmonary epithelium, respectively, and subsequently distributed to various tissues, including fetal tissues. PAHs can be detected in diverse tissues and organs, such as the lungs, skin, esophagus, liver, colon, and placenta. The body attempts to increase their hydrophilicity to facilitate excretion through oxidative metabolism. Benzo[a]pyrene, recognized as the first discovered chemical carcinogen, is employed as an indicator in health risk assessments of PAH mixtures, both in terms of the contamination levels and carcinogenic risk. This selection is based on two primary factors: its heightened carcinogenic potency compared to other PAHs and its consistent concentration ratios with other PAHs, especially carcinogenic ones, within PAH mixtures, depending on the pollution source. Numerous researchers advocate for the biomonitoring of potential PAH exposure to prevent the bioaccumulation of these hazardous substances. The current research primarily focuses on quantifying 1-hydroxypyrene (1-OH-PYR) as a surrogate for total PAH exposure or other hydroxylated PAH metabolites excreted in urine [87,88]. Indeed, urinary PAH metabolites can reflect the internal dose and serve as sensitive biomarkers of exposure [89,90,91].

Other researchers have investigated PAH-DNA adducts in the blood as indicators of the future disease risk [92] or epigenetic alterations in spermatozoa as potential transgenerational effects of environmental toxicants [93]. A limited number of studies have reported the quantification of PAHs in circulating blood or other biofluids using mass spectrometry, suggesting this approach as a promising tool to assess exposure biomarkers in environmental and population studies [94,95].

### 4.2. Impacts of PAHs on Human Health

PAHs have demonstrated toxic, carcinogenic, genotoxic, mutagenic, and teratogenic properties [85,96] and exhibit potent immunosuppressive effects [97]. Low-molecular-weight PAHs (two to three rings) exhibit acute toxicity but lack carcinogenic potential, whereas high-molecular-weight PAHs (four to seven rings) display reduced toxicity but possess carcinogenic, mutagenic, and teratogenic effects in various organisms, including fish, amphibians, birds, and mammals [98,99,100]. The literature suggests that low-molecular-weight PAHs (one to three rings) exhibit high toxicity, while higher-molecular-weight PAHs demonstrate genotoxic effects [101].

Table 2 summarizes the complex data on PAH exposure, health impacts, and symptoms by various sources (e.g., exposure via inhalation, occupational, prenatal, etc.), highlighting both the immediate and long-term health effects and the relevant studies.

The effects of PAHs on human health primarily depend on the method, timing, and extent of exposure, as well as the particular types of PAHs involved. Additional elements, such as age and pre-existing medical conditions, can also modulate the health outcomes. Although it is still unclear exactly how PAHs contribute to immediate health problems in individuals, there have been instances where those exposed to high concentrations of PAHs—such as workers handling polluted materials—have shown symptoms like eye discomfort, nausea, vomiting, diarrhea [78], and disorientation, all of which highlight the acute toxicity of these substances [42]. However, the exact components of the PAH mixtures that cause these health issues are still unknown. It has also been observed that PAH mixtures can irritate the skin, leading to redness and swelling. Chemicals like anthracene, benzo[a]pyrene, and naphthalene are particularly known for causing direct skin irritation [102]. Working with PAHs, especially in industries such as coal tar production, aluminum smelting, and asphalt paving, has been linked to an elevated risk of cutaneous malignancies. Benzo[a]pyrene, for instance, has been implicated in the development of skin tumors in animal models and is classified as a Group 1 carcinogen (carcinogenic to humans) for skin cancer by the IARC [43].

Chronic exposure to PAHs can result in adverse health outcomes. These include damage to the immune system, cataracts, kidney and liver issues; breathing problems; asthma-like symptoms; and reduced lung function. Repeated dermal contact can elicit erythema and inflammation. The ingestion or inhalation of substantial quantities of naphthalene can induce hemolysis, characterized by the destruction of erythrocytes [105]. Further research should investigate the mechanisms of gastrointestinal carcinogenesis induced by exposure to environmental pollutants, such as PAHs [106,107].

Breathing in PAHs can induce respiratory tract irritation, manifesting as symptoms such as coughing, pharyngeal irritation, and dyspnea. PAHs can also elicit airway inflammation, contributing to respiratory pathologies such as asthma and chronic bronchitis [103]. Long-term exposure to PAHs has been associated with diminished pulmonary function parameters, including the forced expiratory volume in one second and the forced vital capacity. This suggests that chronic PAH exposure may contribute to the pathogenesis of respiratory diseases, such as chronic obstructive pulmonary disease. PAH exposure has also been linked to the exacerbation of asthma symptoms in susceptible individuals and reduced pulmonary function in vulnerable populations [103]. Chronic exposure to PAHs has been associated with an elevated risk of respiratory malignancies. The inhalation of PAH-contaminated air pollutants, particularly in urban and industrial environments, has been linked to an increased incidence of lung cancer [104,120,121,122], a leading cause of cancer-related mortality worldwide, accounting for 1.59 million deaths in 2012 [43]. Certain PAHs, notably benzo[a]pyrene, are designated as Group 1 carcinogens (carcinogenic to humans) by the IARC, specifically due to their implications in pulmonary carcinogenesis [103]. A separate review and meta-analysis examined the relationship between PAHs, cardiovascular diseases, and blood pressure [123].

A recent study found that people with the highest levels of naphthalene, fluorene, phenanthrene, and other PAH metabolites in their urine had a much higher risk of developing diabetes compared to those with the lowest levels. This suggests a link between exposure to PAHs and the development of this metabolic condition [108]. Numerous studies demonstrate a direct correlation between parental exposure to polycyclic aromatic hydrocarbons before conception or during pregnancy and the development of acute lymphocytic leukemia in offspring. Fetal exposure to PAHs can occur transplacentally, while postnatal exposure can occur through breastfeeding, the inhalation of contaminated air, the ingestion of contaminated food, or dermal absorption. Several studies indicate that maternal occupational exposure to PAHs can increase the risk of small-for-gestational-age infants, preterm births, and congenital heart defects [110]. Elevated prenatal exposure to polycyclic aromatic hydrocarbons has been associated with detrimental effects on neurodevelopment in children, including reduced intelligence quotients, cognitive impairments, and increased behavioral difficulties by ages six to eight [111,112]. Prenatal maternal exposure to PAHs has been linked to attention deficit/hyperactivity disorder, as evidenced by a meta-analysis demonstrating a significant association between gestational PAH exposure and the ADHD prevalence [113].

Recent studies indicate that PAHs can traverse the blood–brain barrier, exerting neurotoxic effects on the central nervous system. These mechanisms include direct cytotoxicity to neurons and glial cells, potentially contributing to neurodevelopmental disorders in children and cognitive impairments such as memory, attention, and learning deficits. Vulnerable populations, including children and the elderly, exhibit increased susceptibility [109].

Long-term exposure to PAHs has been implicated in cognitive deficits and an elevated risk of neurodegenerative disorders, including Parkinson’s disease and Alzheimer’s disease. Oxidative stress and neuroinflammation are postulated to mediate these effects, although the precise mechanisms remain to be fully elucidated. PAH exposure can elicit neuroinflammatory responses in the brain, potentially triggering microglial activation and the subsequent release of pro-inflammatory cytokines and other mediators. Neuroinflammation is thought to contribute to neuronal damage and may be involved in the pathogenesis and progression of neurological disorders associated with PAH exposure [109].

Epidemiological research indicates a significant association between exposure to polycyclic aromatic hydrocarbons and the development of prostate cancer [114,115]. The WHO stated that prostate cancer was the second most prevalent cancer in men globally, with an estimated 1.1 million diagnoses and 0.3 million mortalities in 2012 [116]. Prostate cancer exhibits a higher incidence in economically developed nations, likely attributable to the increased implementation of early detection screening programs. Established risk factors for this malignancy include advanced age, a family history of prostate cancer, and ethnicity, with African American men demonstrating a two-fold greater risk compared to Caucasian men [124]. Notwithstanding, the precise contribution of environmental and dietary factors, and their associated mechanisms, in the pathogenesis of this neoplasm remains to be fully elucidated.

While the prostate is a target organ for PAH exposure, the breast is also susceptible, as described in a systematic review and meta-analysis by Gamboa-Loira et al. [117], in which the association between PAH and breast cancer was investigated. Several studies confirm the role played by PAH metabolites in developing breast cancer, so PAH exposure can be considered a risk factor.

Multiple studies corroborate the role of PAH metabolites in breast carcinogenesis, suggesting that PAH exposure may constitute a risk factor. A meta-analysis by Rota M et al. [125] confirmed the elevated risk of bladder cancer in individuals with occupational exposure to PAHs. Indeed, workers in industries such as aluminum production and coke ovens have been reported to exhibit an increased risk of bladder cancer. The presence of PAH-DNA adducts in the bladder tissue further substantiates the link between PAH exposure and bladder carcinogenesis [126].

PAHs may also exert deleterious effects on human health in the context of rheumatology and autoimmunity. Xi et al. [118] postulated a link between PAH exposure and rheumatoid arthritis, elucidating the potential pathogenetic role of PAHs in this disease, primarily by exploring the mechanisms of interaction between PAHs and aryl hydrocarbon receptors. Furthermore, PAH exposure has been associated with immunosuppressive effects, potentially interfering with immune system function by suppressing the activity of immune cells, including T cells, B cells, and natural killer cells. This immunosuppression may compromise the immune system’s ability to mount effective responses against infections and neoplastic cells [119].

Furthermore, PAH exposure may also engender increased susceptibility to various infections. Studies indicate that individuals exposed to elevated levels of PAHs may exhibit increased vulnerability to respiratory infections, gastrointestinal infections, and other infectious diseases due to impaired immune function. PAH exposure has also been linked to an elevated risk of allergic and autoimmune diseases. The immunomodulatory effects of PAHs may contribute to the development or exacerbation of conditions such as asthma, allergic rhinitis, and autoimmune disorders, potentially by disrupting the balance between pro-inflammatory and anti-inflammatory responses, leading to dysregulated immune reactions. Moreover, the immunotoxicity of PAHs may compromise the efficacy of vaccinations. Studies have demonstrated that exposure to PAHs may impair the immune response to vaccines, reducing antibody production and diminishing vaccines’ effectiveness, which may have implications for public health and vaccination strategies. The experimental evidence from the aforementioned studies suggests that the effects of PAHs on human health are contingent upon the duration and route of exposure. This underscores the potential hazard to human health posed by PAHs, necessitating strategies to mitigate both environmental and occupational exposure to these pollutants in order to reduce the risk to human health. A more comprehensive risk assessment may be predicated on the data presented herein, but further research is warranted to inform the development of effective mitigation strategies.

### 4.3. Impacts of PAHs on Female Fertility

Beyond the well-established effects of environmental PAH exposure on male reproductive health, accumulating evidence suggests detrimental effects on the female reproductive system as well. Bolden et al. highlighted that PAHs may cause damage to egg cell DNA and disrupt ovarian function, which could contribute to conditions like polycystic ovary syndrome, miscarriage, and preterm births [127]. As previously mentioned, PAHs can exert estrogenic or antiestrogenic effects, acting as endocrine disruptors of reproductive function. Their impact on female fertility has been recognized since 1998, when Zenzes et al. reported PAH-induced DNA adducts in the granulosa cells of women undergoing in vitro fertilization who were exposed to cigarette smoke [128]. Subsequently, they also observed a higher prevalence of these adducts in the embryos of couples who smoked compared to those of non-smoking couples [128,129]. This suggests that PAH-induced DNA damage may be transmitted to the progeny. The mechanisms by which PAHs affect female fertility have become a subject of intense research.

These mechanisms appear to be similar to those affecting male fertility. PAH exposure can induce oxidative stress in the reproductive tissues, including the ovaries and testes. PAHs generate reactive oxygen species during their metabolism, potentially overwhelming the endogenous antioxidant defenses and causing cellular damage. Oxidative stress can impair gamete quality and function, contributing to infertility. PAH metabolites are genotoxic and can form DNA adducts, inducing mutations. DNA damage in gametes can lead to genetic abnormalities in offspring and increase the risks of infertility and adverse pregnancy outcomes.

PAH-induced epigenetic changes in the reproductive tissues may affect gene regulation and cellular functions that are essential for fertility and reproductive success. PAH exposure can also trigger inflammation and immune responses in the reproductive system, further compromising fertility. It has been demonstrated that chemical insults can deplete the primordial follicle pool, adversely affecting fertility [130,131]. Conversely, xenobiotic exposures that induce damage to primary, secondary, and antral follicles may result in transient infertility and anovulation. When PAH exposure affects the entire follicle pool, either temporary infertility or premature ovarian insufficiency may ensue [130]. Benzo[a]pyrene is metabolized via a specific pathway in the ovaries, generating metabolites implicated in the pathogenesis of infertility and cancer [132].

Numerous investigations employing in vivo animal models have suggested that PAHs exert deleterious effects on ovarian follicles, oocytes, and cumulus–oocyte complexes via mechanisms analogous to those described for spermatozoa [133,134].

PAHs present in the follicular fluid of women exposed to cigarette smoke undergoing in vitro fertilization negatively affect ovarian germ cells [95,135]. Numerous in vivo and in vitro studies have explored the association between PAH exposure and ovarian germ cell apoptosis [136,137,138]. Exposure to benzo[a]pyrene disrupts mouse oocyte meiotic progression by interfering with normal spindle assembly, chromosome alignment, and kinetochore–microtubule attachment, resulting in the production of aneuploid eggs. Consequently, benzo[a]pyrene exposure diminishes female fertility by impairing oocyte maturation [139]. Furthermore, environmental PAH exposure is correlated with alterations in endocrine markers of ovarian function in women, exhibiting PAH-specific patterns [140].

Another compelling aspect of fertility is the influence of PAHs on infertility in couples. Numerous studies have investigated populations of couples undergoing fertility treatment and IVF cycles. For instance, a small-scale, monocentric cohort study by Netter et al. [141] compared PAH exposure between couples with positive versus negative human chorionic gonadotropin results 14 days after embryo transfer. Their findings revealed that the urinary 1-hydroxypyrene (1-OH-PYR) levels were significantly lower in women with positive HCG tests compared to those with negative results. The urinary 1-OH-PYR levels in women correlated with embryo fragmentation in the highest-quality embryos, suggesting a relationship between PAH exposure and reduced embryo quality. Moreover, the urinary concentrations of hydroxylated PAHs, particularly 2-hydroxyphenanthrene plus 3-hydroxyphenanthrene (2 + 3 PHE), were positively associated with early pregnancy loss in women undergoing in vitro fertilization–embryo transfer (IVF-ET) [142].

### 4.4. Impacts of PAHs on Male Fertility

A growing body of scientific literature demonstrates the detrimental effects of PAHs on male fertility. Numerous studies indicate that PAH exposure can negatively impact the reproductive capacity of male subjects [47,143,144]. Investigations utilizing both human and animal models have explored the potential correlation between PAH exposure and male reproductive function [145,146,147,148,149].

Table 3 provides a summary of the effects of PAH exposure on reproductive health, focusing on the health impacts, symptoms, and references from the literature.

A correlation between PAH exposure and sperm DNA damage, as well as sperm dysfunction, in human males has been recently reported [150]. Fifty-four non-smoking male participants were selected for semen analysis, which included assessments of the semen volume, sperm concentration, morphology, motility, acrosome reaction, plasma membrane integrity, DNA damage (via sperm chromatin structure assay—SCSA), and sperm motion characteristics (via computer-assisted semen analysis—CASA). The study demonstrated a seasonal variation in the semen parameters and sperm quality, closely correlated with fluctuations in the ambient pollutant concentrations. An inverse association between maternal smoking during pregnancy and the total sperm count (*p* = 0.002) was also observed. Men whose mothers smoked more than 19 cigarettes per day during pregnancy exhibited reductions in their semen volume (approximately 19%, *p* = 0.04), total sperm count (approximately 38%, *p* = 0.11), and sperm concentration (approximately 17%, *p* = 0.47) compared to unexposed men [164]. These findings indicate that prenatal exposure to PAHs from tobacco smoke adversely affects male sperm quality, potentially contributing to the observed population differences and temporal trends in semen quality. Furthermore, smoking is associated with an elevated risk of erectile dysfunction, while smoking cessation appears to correlate with the restoration of erectile function [165]. Notably, the authors do not assert that the observation of adverse effects from tobacco smoking or air pollution implies causality solely attributable to PAHs. However, numerous human health effects have been documented following PAH exposure through both cigarette smoking and food processing. Xia et al. demonstrated a positive association between elevated urinary concentrations of 1-hydroxypyrene, 2-hydroxyfluorene, and PAH metabolites and an increased risk of idiopathic male infertility [151]. A crucial concern is that PAH-induced infertility may not be discernible through conventional semen analysis [129,166].

Contemporary research has increasingly emphasized the exposome, encompassing the totality of environmental exposures and individual experiences throughout the lifespan. Nayak et al. [152] investigated the relationship between the seminal PAH exposome and sperm function in idiopathic male infertility by analyzing spermatozoa proteins and gene expression. They concluded that seminal PAH concentrations, along with oxidative protein modifications and the expression of the aryl hydrocarbon receptor and heat shock protein 90-beta (HSP90-B), could serve as biomarkers in differentiating between idiopathic infertile and fertile men. Elevated PAH concentrations were observed in the urine of the selected infertile groups, suggesting a strong correlation between PAH exposure and male infertility. Several mechanisms are thought to contribute to the detrimental effects of PAHs on fertility. One such mechanism is DNA methylation, which has been explored in recent studies analyzing a cohort of policemen and their exposure to air pollution [153,154]. Epigenetic alterations can modify gene expression profiles without altering the underlying DNA sequence. PAH-induced epigenetic changes in sperm may impact fertility and contribute to reproductive health problems. One potential mechanism is the action of PAH metabolites as endocrine-disrupting compounds (EDCs), a class of chemicals that can interfere with both hypothalamic–pituitary–gonadal axis hormones and testicular hormones [155]. Strong support for this perspective comes from the European Association of Urology, which has attributed idiopathic male factor infertility to endocrine disruption resulting from reactive oxygen species, genetic anomalies, and environmental contamination [156].

These compounds have been demonstrated to interact with hormone receptors and disrupt the synthesis and signaling pathways of reproductive hormones, including testosterone and follicle-stimulating hormone [157]. Certainly, several studies have indicated that these compounds can emulate endogenous hormones [158]; for instance, their metabolites may exhibit estrogenic activity [167] or disrupt thyroid function [168], thereby affecting the hypothalamic–pituitary–thyroid axis and hypothalamic–pituitary–gonadal axis. However, the precise mechanism by which PAHs exert their toxic effects on male fertility remains elusive. Some researchers have proposed that PAHs may bind to and activate the aryl hydrocarbon receptor, leading to the increased metabolism of PAHs into DNA-reactive products [159]. Studies have shown that human sperm expresses aryl hydrocarbon receptor and aryl hydrocarbon receptor nuclear translocator mRNA, suggesting a potential impact on sperm function [169].

Reactive metabolites generated by cytochrome P450 enzymes induce the production of reactive oxygen species (ROS) [160], which can cause DNA oxidation or the formation of PAH-DNA adducts. These adducts are recognized as indicators of sperm genotoxicity and male factor infertility [161,166]. Elevated levels of ROS can suppress steroidogenesis and induce mitochondrial membrane degeneration in spermatozoa [162]. An alternative mechanism involves the formation of PAH epoxide metabolites via a cytochrome P450-dependent monooxygenase system. The overexpression of this system can augment the generation of reactive oxygen species, leading to DNA damage [163,170]. Certainly, oxidative stress is associated with diminished sperm function and is a contributing factor to male infertility [171,172,173].

The findings of the aforementioned studies demonstrate a substantial inverse correlation between PAH metabolites and sperm parameters (concentration, volume, motility, morphology), as well as DNA integrity, suggesting a link between PAH exposure and male factor infertility. Based on expert opinion, further investigation is warranted to elucidate the precise relationship between PAH exposure and adverse reproductive outcomes in humans, particularly in occupational settings. Many studies have measured the PAH concentrations in biological fluids (e.g., semen), without accounting for the potential contributions of other pollutants to the observed detrimental effects.

## 5. Conclusions

PAHs are ubiquitous environmental contaminants with significant human health implications, especially in occupational environments. Evidence suggests that PAHs pose substantial health risks, including carcinogenic, mutagenic, and teratogenic effects, which are of particular concern in industries with high exposure levels. Moreover, the potential impact of PAHs on reproductive health is increasingly recognized, with research indicating their involvement in endocrine disruption, oxidative stress, and DNA damage. These effects underscore the importance of the stringent monitoring and regulation of PAH emissions and exposures. To mitigate the PAH-associated health risks, comprehensive workplace safety measures are essential, including adequate ventilation, personal protective equipment, and routine health surveillance for exposed workers. Furthermore, reducing PAH emissions from industrial and vehicular sources through technological advancements and cleaner energy alternatives is crucial. Public awareness campaigns and education regarding the hazards of PAHs, combined with robust regulatory frameworks, can help to minimize exposure and safeguard public health. Future research should prioritize understanding the long-term health consequences of PAH exposure and developing novel strategies for detection, prevention, and treatment. Addressing PAH contamination necessitates a multi-pronged approach involving collaboration between industry, governments, and the public. These findings highlight the broader impact of environmental exposures on reproductive health, emphasizing the need for a comprehensive strategy to reduce PAH exposure and protect both male and female fertility.

## Figures and Tables

**Table 1:** List of sixteen molecules classified as “priority pollutants” by the US-EPA *.

PAH	Structure	Formula	Molecular Weight g/mol	Melting Point °C	Boiling Point °C	Solubility
Acenapthene	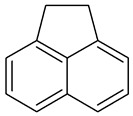	C_12_H_10_	154.2	93.4 °C	279 °C	Anhydrous acid benzene, chloroform, petroleum ether, and toluene
Acenapthylene	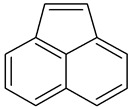	C_12_H_8_	152.2	78–82 °C	280 °C	Benzene, chloroform, dietheyl ether, and EtOH
Anthracene	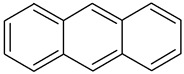	C_14_H_10_	178.2	218 °C	340 °C	MeOH and hexane
Benz(a)anthracene	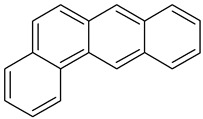	C_18_H_12_	228.3	158 °C	438 °C	Acetone, diethyl ether, and 0.00001 g/100 mL in water
Benzo(a)pyrene 1	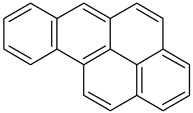	C_20_H_12_	252.3	179 °C	495 °C	Benzene, toluene, xylene, and sparingly soluble in alcohol
Benzo(b)fluoranthene 2B	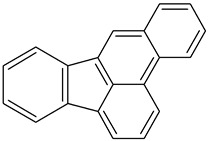	C_20_H_12_	252.3	168°	228.6°	Acetone, alcohol, and benzene
Benzo(g,h,i)perylene	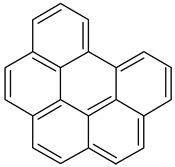	C_22_H_12_	276.3	278°	500°	Acetone, dichloromethane, and 1,4 dioxane
Benzo(k)fluranthene 2B	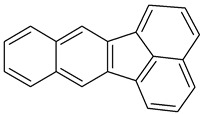	C_20_H_12_	252.3	217°	228.6°	Benzene
Chrysene 2B	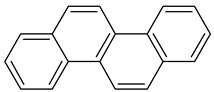	C_18_H_12_	228.3	254°	448°	Ethanol
Dibenz(a,h)anthracene 2A	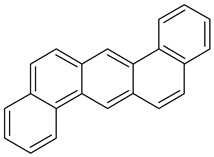	C_22_H_14_	278.3	262°	524°	Benzene, ether, petroleum, toluene, xylene, and slightly soluble in alcohol and ether
Fluranthene	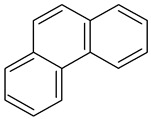	C_16_H_10_	202.26	375°	110.8°	Acetic acid and hot alcohol, benzene, ethanol, ethyl ether
Fluorene	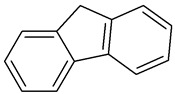	C_13_H_10_	166.223	295°	116–117°	Benzene and ether
Indeno [1,2,3-cd]pyrene 2B	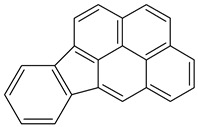	C_22_H_12_	276.337	162–164°	497.101°	Benzene
Napthalene	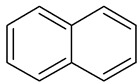	C_10_H_8_	128.17	218°	80.26°	Very soluble in carbon disulfide, chloroform, and ether; less soluble in ethanol/methanol; and insoluble in water
Phenanthrene	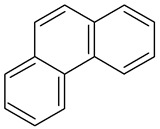	C_14_H_10_	178.23	101°	332°	Insoluble in water. Soluble in most organic solvents, such as acetic acid, benzene, carbon tetrachloride, chloroform, ether, and toluene
Pyrene	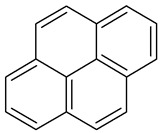	C_16_H_10_	202.25	145–148°	404°	Soluble in benzene, ethanol, and ether

* 1 = proven carcinogen, 2A = probable carcinogen, and 2B = possible carcinogen.

**Table 2:** Summary of PAH exposure sources, health impacts, symptoms, and relevant studies on immediate and long-term effects.

Source	Human Health Impacts	Symptoms	References
Low-molecular-weight PAHs (2–3 rings)	Acute toxicity; lack of carcinogenic potential.	Immediate symptoms like eye discomfort, nausea, vomiting, diarrhea, disorientation.	[85,96]
High-molecular-weight PAHs (4–7 rings)	Carcinogenic, mutagenic, and teratogenic properties; reduced acute toxicity but exhibit harmful effects in organisms.	Symptoms of long-term exposure such as skin irritation, respiratory issues, and cancer development in certain organs.	[98,99,100,101]
Occupational Exposure (e.g., coal tar, aluminum smelting, asphalt)	Skin cancer risk, immunosuppressive effects, potential for cutaneous malignancies, lung cancer, respiratory diseases, and cardiovascular disease.	Skin irritation, redness, swelling, coughing, dyspnea, lung cancer, immune suppression, reduced lung function.	[42,43,102,103,104]
Chronic exposure (e.g., inhalation, ingestion)	Respiratory irritation, asthma, bronchitis, neurodegenerative diseases (Parkinson’s, Alzheimer’s), gastrointestinal and immunological damage, cardiovascular diseases.	Coughing, breathing problems, fatigue, neurodegeneration, reduced lung function, gastrointestinal distress.	[105,106,107,108,109]
Prenatal exposure (maternal exposure)	Fetal neurodevelopmental issues, cognitive impairments, behavioral difficulties, ADHD, congenital heart defects, increased risk of childhood leukemia, and small-for-gestational-age babies.	Reduced IQ, behavioral issues, developmental delays, attention deficit/hyperactivity disorder, preterm birth, congenital defects.	[110,111,112,113]
PAHs crossing blood–brain barrier	Neurotoxic effects, potentially contributing to developmental disorders and cognitive impairments.	Memory, attention, and learning deficits; potential for neurodevelopmental disorders.	[109]
Prolonged PAH exposure (including occupational)	Increased risks of prostate cancer, bladder cancer, and breast cancer, associated with metabolic conditions like diabetes and other cancers.	Cancer symptoms, metabolic disturbances like increased risk of diabetes, cognitive deficits.	[114,115,116,117]
Immunosuppressive effects (PAH exposure)	Weakening of immune system, increased susceptibility to infections, autoimmune diseases (e.g., rheumatoid arthritis), and diminished vaccine efficacy.	Increased vulnerability to respiratory and gastrointestinal infections, fatigue, skin conditions, weakened immune response.	[118,119]

**Table 3:** Effects of PAH exposure on male and female reproductive health: health impacts, symptoms, and relevant literature.

Source	Human Health Impacts	Symptoms	References
PAH exposure (female reproductive system)	Damage to egg cell DNA, disruption of ovarian function, conditions like polycystic ovary syndrome, miscarriage, preterm birth, endocrine disruption, reduced fertility.	Infertility, miscarriage, hormonal imbalances, polycystic ovary syndrome, disrupted menstrual cycles.	[127,128,129]
PAH-induced DNA adducts in females	DNA damage in oocytes, embryo quality impairment, genetic abnormalities, possible transgenerational effects.	Reduced embryo quality, potential for genetic abnormalities in offspring, impaired oocyte maturation.	[128,129,135]
Oxidative stress in female reproductive tissues	Oxidative stress in ovaries and other reproductive tissues, impaired gamete quality, infertility.	Reduced egg quality, difficulty conceiving, impaired reproductive function.	[130,131,132]
Benzo[a]pyrene exposure (female fertility)	Impaired oocyte maturation, disrupted meiotic progression, chromosome alignment issues, possible infertility.	Impaired egg maturation, reduced fertility, aneuploidy in eggs, fertility problems.	[132,139]
PAH exposure (male reproductive system)	Decreased sperm quality (DNA damage, dysfunction), infertility, impaired semen parameters, oxidative stress, DNA methylation, endocrine disruption.	Reduced sperm count, motility, concentration, and morphology; erectile dysfunction.	[47,143,144,145,146,147,148,149]
PAH exposure and sperm DNA damage	PAH-induced sperm DNA damage, decreased sperm quality, possible infertility.	Reduced sperm motility, concentration, and morphology; increased DNA damage in sperm.	[150,151]
Urinary PAH metabolites in male infertility	Elevated urinary PAH metabolites associated with idiopathic male infertility, disrupted sperm function, oxidative protein modifications.	Impaired sperm function, lower sperm count, and motility.	[151,152]
PAH-induced epigenetic alterations (male)	Epigenetic modifications in sperm DNA, impaired fertility, potential for transgenerational effects.	Altered gene expression in sperm, reduced fertility potential, genetic instability.	[153,154,155]
PAH exposure (endocrine disruption in males)	Endocrine disruption via interference with reproductive hormones, including testosterone and follicle-stimulating hormone, potentially affecting sperm function.	Disrupted hormone levels, reduced testosterone, impaired sperm production.	[156,157,158,159]
Reactive oxygen species and PAH exposure (male)	Increased ROS production in sperm leading to DNA oxidation, sperm genotoxicity, infertility, mitochondrial degeneration, and diminished sperm function.	Reduced sperm motility, compromised DNA integrity, mitochondrial damage.	[160,161,162,163]
PAH exposure in occupational settings (male)	Long-term PAH exposure in occupations linked to increased male infertility risk, decreased sperm parameters, potential genetic mutations.	Reduced sperm concentration, morphology, motility, and genetic integrity; possible infertility.	[152,160,163]
